# Resection of a Portion of the Continuity of the Ulna and Radius for Ununited Colles Fracture

**Published:** 1875-04

**Authors:** 


### Resection of a portion of the Continuity of the Ulna and Radius for the Correction of Deformity from Ununited Colles's Fracture.

Dr. William A. Byrd, of Quincy, Illinois, describes (*Richmond and Louisville Med. Journal*, Oct., 1874), the following interesting case which resembles in many respects one lately reported by Mr. Annandale (see *Monthly Abstract of Medical Sciences* for March, 1875, page 127).

In the summer of 1873, Dr. Byrd was consulted by a clerk, aged 41, as regards the advisability of an attempt to correct a deformity of the left arm. On examination of the limb there was found an ununited Colles's fracture of the left radius. The ulna passed up alongside of the hand until the lower end of it was somewhat beyond the junction of the fifth metacarpal bone and the carpus, and curved at a point opposite the fracture in the radius, the concavity looking toward the radius. The hand was smaller than its fellow, with palm cupped and the fingers partially flexed, with an inability to close them completely or with any degree of force. When he would pronate or supinate the hand, it would describe an arc of the circumference of a cone, the apex of which would be the lower end of the ulna—a very uncouth motion, indeed.

With the belief that he could remedy the defect to a great extent, Dr. Byrd determined to operate on Nov. 1. He made an incision through the skin over the inner side of the ulna, commencing an inch above the styloid process, and continued up the arm for about three inches. The superficial fascia lying at the bottom of the wound, was then very carefully divided over a grooved director. The muscles were pressed apart, so as to expose the bones. The periosteum was divided for an inch and a half, and separated from the whole circumference of the bone; a chain-saw was then passed around the bone at the lower angle of the wound, and the bone quickly divided. The lower end of the upper piece of the ulna was then raised on a spatula, and an inch of it sawed off. An incision two inches long was then made over the point of fracture of the radius, on the outer side of the arm, down to the bone, the ligamentous union between the fragments was broken up with some difficulty. An effort was then made to bring the ends of the fragments through the wound at the radial side, but failing in this, they were forced out through the wound on the ulnar side, and their ends trimmed with a pair of bone nippers. A hole one-sixteenth of an inch in diameter was then drilled from the outer surface of the upper and lower portions of the ulna, commencing one-fourth of an inch from the point of section, and passing diagonally towards the free ends into the medullary cavity. A well annealed iron wire was then passed through the holes and the ends of the wire twisted together, so as to bring the ends of the bones nicely into apposition; the wire was then cut off, leaving a short piece of the twist, which was bent down parallel with the shaft of the bone to prevent irritation of the soft parts.

The ends of the radius being in apposition were left without further interference. The wounds were closed with interrupted sutures and a straight splint applied to the arm anteriorly.

*Nov.* 20. Radial wound healed; ulnar wound healed throughout the greater portion of its extent. An abscess had formed on the palmar aspect of the arm at a point midway between the incisions. When opened, a considerable amount of pus was discharged. This continued to discharge until Feb. 7, 1874, when probing it Dr. Byrd discovered a foreign substance at the bottom of the sinus. Enlarging the opening, he was enabled to extract the wire with two bits of bone that had been included in it in tying the two pieces of ulna together. At this time the ulna had slipped up so as to occupy an almost normal position with the carpus, and the radius had united at the point of fracture; the ulna was still ununited. There was also a small opening at the ulna wound, through which water would pass when injected into the opening on the front of the arm. After this, these openings rapidly healed, and the ulna firmly united. He had some degree of pronation and supination, with good use of the wrist. The fingers became more flexible and the hand less cupped and deformed.—*Monthly Abstract Medical Sciences.*

